# Hepatitis B Virus Impairs TLR9 Expression and Function in Plasmacytoid Dendritic Cells

**DOI:** 10.1371/journal.pone.0026315

**Published:** 2011-10-25

**Authors:** Isabelle E. Vincent, Claudia Zannetti, Julie Lucifora, Helene Norder, Ulrike Protzer, Pierre Hainaut, Fabien Zoulim, Massimo Tommasino, Christian Trépo, Uzma Hasan, Isabelle Chemin

**Affiliations:** 1 INSERM, U871, Lyon, France; 2 Université Lyon 1, Lyon, France; 3 INSERM, U851, Faculte de Medicine Lyon Sud, Pierre Benite, France; 4 Hospices Civils de Lyon, Lyon, France; 5 Institute of Virology, Technische Universität München, Helmholtz Zentrum München, Munich, Germany; 6 Virological Department, Swedish Institute for Infectious Disease Control, Solna, Sweden; 7 International Agency for Research on Cancer, Lyon, France; Albany Medical College, United States of America

## Abstract

Plasmacytoid dendritic cells (pDCs) play a key role in detecting pathogens by producing large amounts of type I interferon (IFN) by sensing the presence of viral infections through the Toll-Like Receptor (TLR) pathway. TLR9 is a sensor of viral and bacterial DNA motifs and activates the IRF7 transcription factor which leads to type I IFN secretion by pDCs. However, during chronic hepatitis B virus (HBV) infection, pDCs display an impaired ability to secrete IFN-α following *ex vivo* stimulation with TLR9 ligands. Here we highlight several strategies used by HBV to block IFN-α production through a specific impairment of the TLR9 signaling. Our results show that HBV particle internalisation could inhibit TLR9- but not TLR7-mediated secretion of IFN-α by pDCs. We observed that HBV down-regulated TLR9 transcriptional activity in pDCs and B cells in which TLR9 mRNA and protein levels were reduced. HBV can interfere with TLR9 activity by blocking the MyD88-IRAK4 axis and Sendai virus targeting IRF7 to block IFN-α production. Neutralising CpG motif sequences were identified within HBV DNA genome of genotypes A to H which displayed a suppressive effect on TLR9-immune activation. Moreover, TLR9 mRNA and protein were downregulated in PBMCs from patients with HBV-associated chronic hepatitis and hepatocellular carcinoma. Thus HBV has developed several escape mechanisms to avoid TLR9 activation in both pDCs and B lymphocytes, which may in turn contribute to the establishment and/or persistence of chronic infection.

## Introduction

Chronic HBV infection affects more than 400 million people worldwide and is a major risk factor for developing liver cirrhosis and hepatocellular carcinoma [1], [2]. Infection outcome relies on the immune system maturity, and approximately 10% of immunocompetent adults will develop a chronic infection, which in 10-30% of cases will progress towards liver disease. The mechanisms involved in viral clearance and persistence still remain elusive [3]. Several studies report that HBV has developed evasion strategies that target the adaptive immune responses [3], [4], but many issues of innate immunity during HBV infection remain to be investigated. Interestingly, the early weeks after infection with undetectable viremia, followed by high levels of replication, are not linked to any detectable activation of type I interferons (IFN-α/β) [5], a key anti-viral response. Plasmacytoid dendritic cells (pDCs) are important contributers in host defence as they produce large amounts of type I IFNs, a critical early step for the innate immune system to control viral replication [6]. The innate immune response plays an essential role in recognizing microbial motifs through a panel of pattern recognition receptors (PRR). The most studied family is the Toll-Like Receptors (TLR), ten TLRs have been identified in Humans [7]. TLR2 and TLR4 recognize Gram-positive and Gram-negative bacteria cell wall products respectively. TLR5 recognizes a structural epitope of bacterial flagellin, whilst TLRs 3, 7 and 8 have been demonstrated to recognize different forms of viral RNA. TLR9 recognises DNA sequences from bacteria or viruses in the form of unmethylated CpG motifs. In humans, TLR9 expression is restricted to pDCs and B lymphocytes among mononuclear cells [7]. Upon TLR9 stimulation mediated by distinct classes of CpG oligonucleotides (CpG ODN), pDCs are activated to produce IFN-α/β and various chemokines, and B lymphocytes are induced to proliferate and secrete IgM and IL-6 [7]. Despite a narrow expression of TLRs, i.e. TLR7 and TLR9, pDCs are specialized in virus recognition. Furthermore, activation of pDCs has a strong impact on conventional DCs, B, T and NK cells, linking innate and adaptive immunity through pivotal events [8]. Importantly, functional impairment of pDCs have been described in several chronic viral diseases induced by human immunodeficiency virus (HIV), hepatitis C virus (HCV) and HBV infections [9]. During acute and chronic HBV infection, reduced numbers of pDCs have been described in some [10], [11], [12] but not all [13], [14] patient studies. In this setting, pDCs display a reduced ability to secrete IFN-α in response to *ex vivo* TLR9 stimulation (HSV-1 or CpG ODN) [10], [12], [13], [15]. In HBV-infected patients, massive quantities of subviral particles (spheres and filaments) containing only envelope antigens (HBsAg) are produced [3] and uptake of large amounts of HBsAg by pDC has been shown to impair *in vitro* TLR9-induced IFN-α secretion [16]. Moreover, recent reports are highlighting specific interactions of HIV, HBV, HCV, Epstein Barr virus (EBV) and Human Papillomavirus (HPV) with regulatory molecules that impair TLR9 signaling [17].

In this study, we investigated the specific mechanisms leading to impairment of the TLR9 pathway. Here, we explored the ability of HBV virions to modulate human pDCs and PBMCs ability to secrete IFN-α. We also observed for the first time that B cell expression and function of TLR9 is suppressed by HBV stimulation.

## Results

### HBV does not trigger IFN-α secretion by pDCs and PBMCs despite efficient internalisation

**Figure 1:**
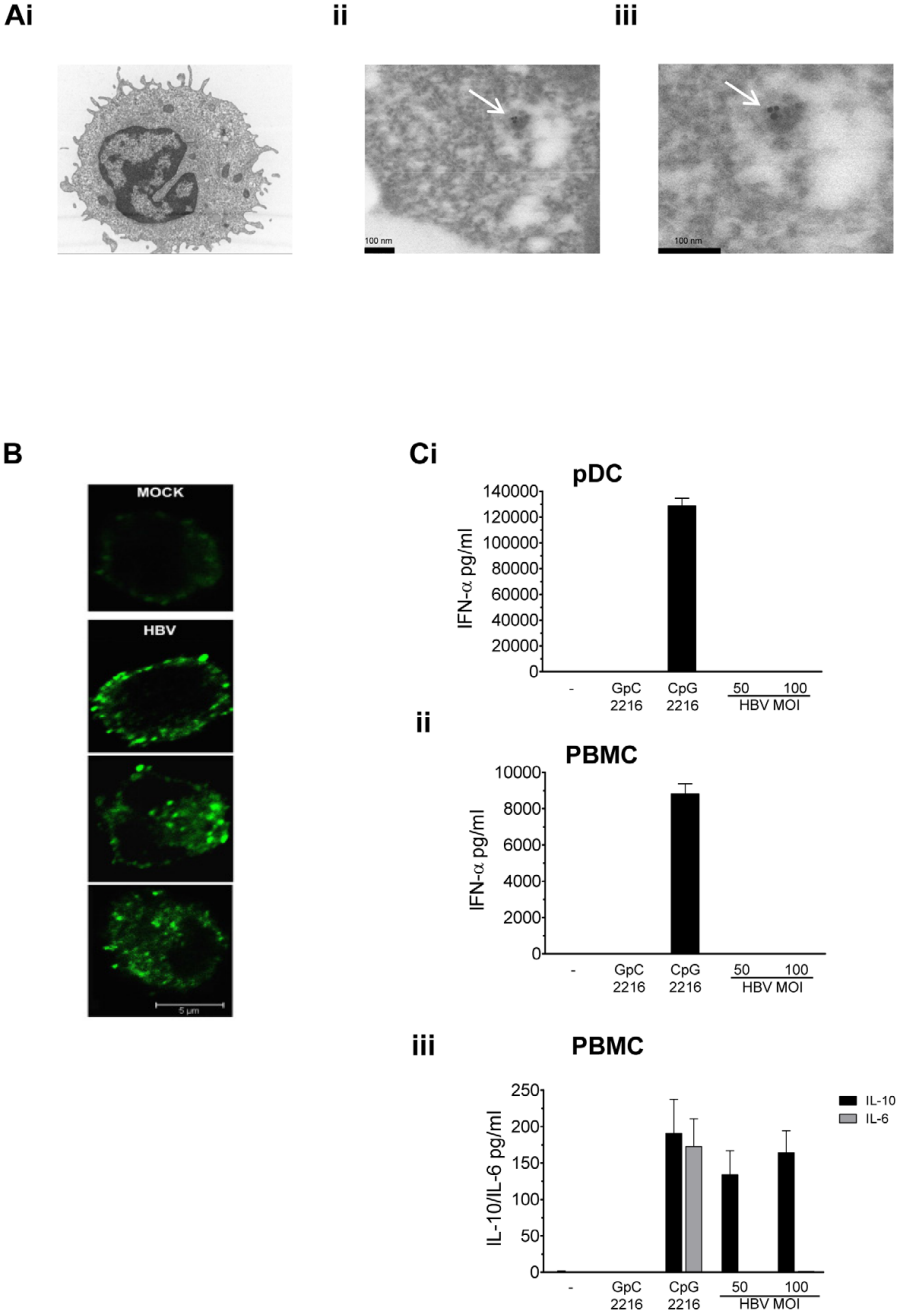
HBV does not induce IFN-α and IL-6 in pDCs despite efficient viral and subviral particles internalisation. (A) Internalisation of HBV virions and subviral particles in pDCs. Freshly isolated pDCs were exposed to HBV at MOI 50 for 1h before cell preparation for immunogold staining (anti-HBsAg Ab) and electron microscopy. HBV Dane particule of 42-45nm diameter (Aii-iii) in an endosomal-like structure with pDC(Ai). Magnification and scale bars Ai x7000, 200nm; Aii x150000, 100nm; Aiii x250000, 100nm. Images are representative of two independent blood donors, 50 cells examination per donor. (B) HBsAg staining in pDCs following HBV exposure. Freshly isolated pDCs were exposed to mock lysate or HBV for 9h at MOI 50 prior to fixation and permeabilization for confocal microscopy analysis. HBsAg staining was detected with rabbit polyclonal anti-HBsAg Ab and Alexa-488 (green) as secondary Ab. Magnification and scale bar, x63; 5 µm. Images are representative of two independent blood donors. (C) HBV does not induce IFN-α secretion in pDCs and PBMC. Freshly isolated pDCs (Ci) and total PBMC (Cii-iii) were exposed to mock lysate or HBV at indicated MOI (GEq/cell). Cells were treated with CpG 2216 at 2µM as a positive IFN-α inducer and GpC2116 as a negative control. Supernatants were collected after 24h and assessed for the presence of IFN-α (Ci-ii), IL-6 and IL-10 (iii) using ELISA. All experiments have been performed using >3 batches of HBV virus and > 3 blood donors. Shown graphs are representative of 1 of 5 experiments that gave similar results.

A previous study on the e*x vivo* analysis of pDCs from highly viremic HBV carriers suggested a selective uptake of subviral spherical HBV particles[18]. To assess the ability of pDCs to internalize both HBV virions and subviral particles, pDCs from healthy individuals were exposed to HBV at MOI 50 for 1h and cells were analysed by electron microscopy (Figures 1A). The specificity of the internalisation was determined by immunogold labeling using anti-HBsAg antibodies (Figure 1Ai-iii). Anti-HBsAg staining allowed the identification of HBV Dane particles (42-45nm, Figure 1Aii-iii) within endosomal structures in pDCs (Ai) as early as 1h post-exposure. Confocal microscopy was also performed after 9h of HBV exposure to assess the intensity of viral and subviral internalisation using anti-HBsAg antibodies (Figure 1B). Intense HBsAg staining, reflecting mostly subviral antigen uptake was observed in pDCs, demonstrated by a diffuse intracytoplasmic or membrane-associated HBsAg staining.

To determine whether HBV could induce a type I IFN response on immune cells, freshly purified human pDCs and PBMC from naïve individuals were exposed to HBV virions for 24h at various multiplicity of infection (MOI), defined as genome equivalent per cell (GEq/cell) (Figure 1Ci and ii). Viral antigens HBsAg and HBeAg were detected within the HBV inoculum by ELISA (data not shown). We observed that HBV did not induce detectable levels of IFN-α neither in purified pDCs nor in PBMC as measured by ELISA (Figure 1Ci and ii). Moreover, immune complexes generated by incubating HBV virions with anti-HBs antibodies did not trigger IFN-α secretion in pDCs (data not shown). As a control, we stimulated both pDCs and PBMC with the CpG oligonucleotide 2216 (CpG 2216), known to activate a TLR9-dependent type I IFN response. Following HBV exposure, moderate levels of IL-10 were induced in PBMC while IL-6 remained below detectable levels (Figure 1Ciii). In summary, despite an efficient internalisation of HBV virions and subviral particles within pDCs, no IFN-α secretion but a moderate IL-10 secretion was observed.

### HBV impairs CpG-induced IFN-α in pDCs and PBMCs

**Figure 2:**
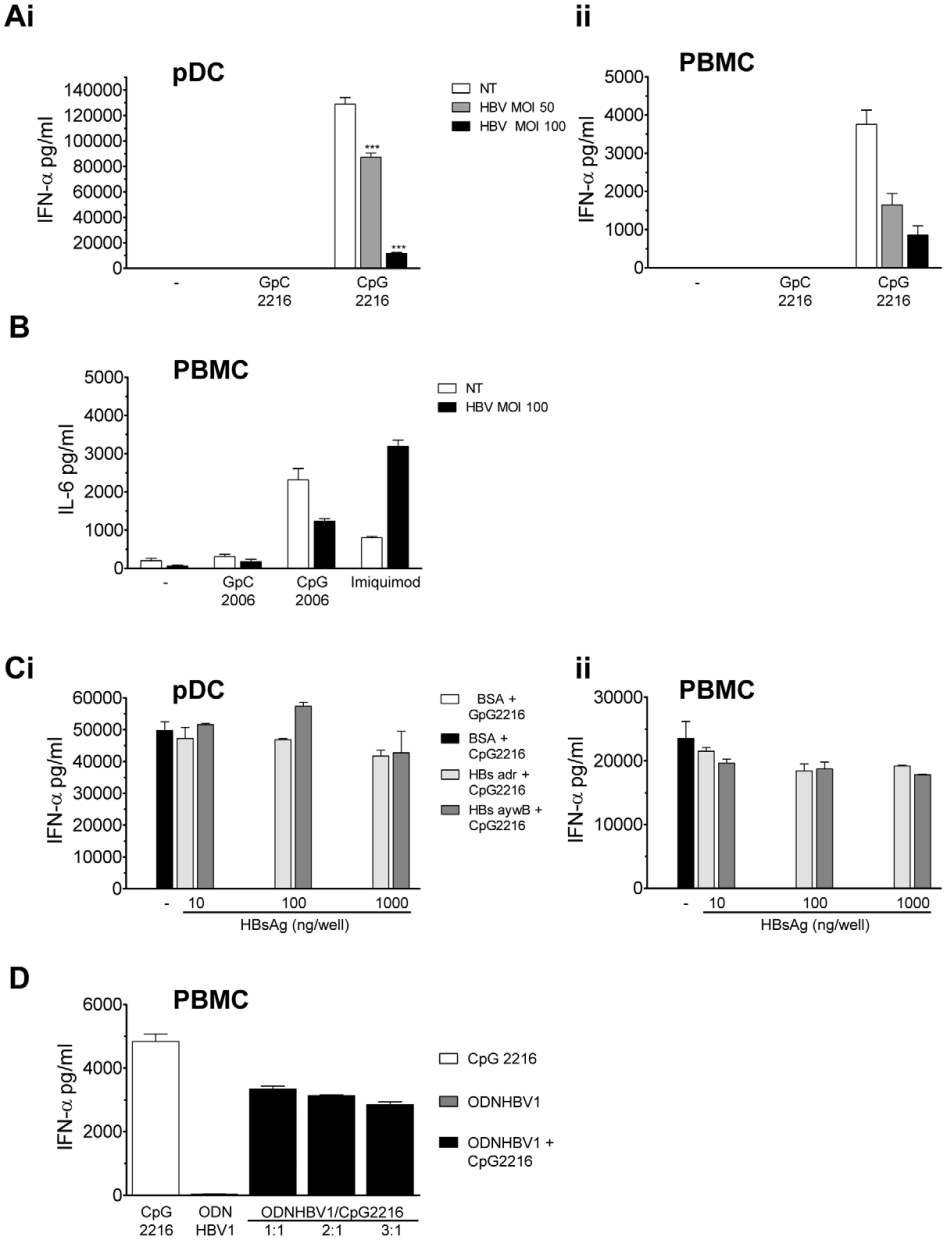
TLR9 functionality is impaired in pDCs and PBMCs in the presence of HBV. (A) HBV impairs TLR9-mediated IFN-α induction in pDCs and PBMC. Purified pDCs (Ai) or total PBMC (Aii) were treated with CpG 2216 (or control GpC 2216) at 2µM ± HBV at the indicated MOI for 24h. Supernatants were tested for IFN-α (pg/ml) using ELISA. (B) HBV impairs TLR9-mediated IL-6 in PBMC. Total PBMC were treated with the indicated TLR ligands ± HBV (MOI 100) for 24h. Supernatants were tested for IL-6 (pg/ml) using ELISA. (C) HBsAg does not suppress CpG-induced IFN-α. Purified pDCs (Ci) or total PBMC (Cii) were stimulated with CpG 2216 or control GpC2216 (2µM) ± increasing concentrations of recombinant HBsAg, adr (clear grey histogram) or ayw3 subtype (dark grey histogram). Supernatants were collected at 24h and analysed for IFN-α (pg/ml) using ELISA. Experiments were performed on cells isolated from >3 different blood donors and graphs are representative of 1 of 5 experiments that gave similar results. (D) An identified HBV-derived ODN sequence inhibits TLR9-mediated IFN-α secretion. PBMC were stimulated with CpG 2216 at 2µM in presence of increasing concentrations of ODN hbv1 (ratio 1:1, 2:1 and 3:1) for 24h and IFN-α secretion was determined in supernatants using ELISA. Experiments are representative of >3 independent blood donors.

We next investigated the consequences of viral internalisation on pDC function. pDCs or total PBMC were stimulated with CpG 2216 in presence of HBV virions at MOI 50 and 100 for 24h (Figure 2Ai and ii). CpG-induced IFN-α was significantly reduced in presence of HBV virions (unpaired student *t* test compared with NT, p<0,001) and inhibition was dose-dependent in both pDCs and PBMC. IFN-α secretion was reduced by 35% in pDCs and by 55% in PBMC at MOI 50. More strikingly at MOI 100, HBV reduced IFN-α secretion in pDCs by 91% and in PBMCs by 75%. HBV could suppress CpG-induced IFN-α as early as 9h following stimulation (data not shown). Although HBV is not a cytopathic virus [4], pDCs viability after 12h of CpG stimulation in the presence of HBV was assessed using annexin V by flow cytometry (Supplementary figure S1). No difference was observed in the rate of annexin V-positive pDCs comparing mock- and HBV-treated cells. Thus, HBV ability to suppress IFN-α secretion in pDCs is not caused by induction of cell apoptosis in our experimental conditions. Since human pDCs express TLR7 and TLR9, we next tested whether HBV could also impair TLR7-induced IFN-α in pDCs using loxoribin (TLR7 ligand) or resiquimod/R848 (TLR7/8 ligand) (supplementary Figure S2A and B) at MOI 50 or 100 for 24h. In contrast to the inhibition observed following TLR9 stimulation, HBV exerted no effect on TLR7-mediated IFN-α secretion (Supplementary figure S2A and B). We also observed that HBV virions at MOI 100 were able to inhibit TLR9-mediated production of IL-6 by CpG 2006 (targeting TLR9 in B cells [7]), but not TLR7 induction of IL-6 by imiquimod (Figure 2B). Interestingly, TLR7-mediated IL-6 production was increased in the presence of HBV virions.

To address a possible involvement of HBsAg in the suppression of IFN-α secretion [16], purified pDCs and PBMC were stimulated for 24h with CpG in the presence of increasing amounts of recombinant HBsAg (adr or ayw3 subtypes (Figure 2Ci and ii)). CpG-induced IFN-α was neither impaired in pDCs nor in PBMC exposed up to 1µg of HBsAg, data which were confirmed using recombinant HBsAg of bacterial origin (data not shown). HBsAg alone did not induce detectable levels of IFN-α, IL-6 or IL-10 in pDCs or PBMC (data not shown). Thus, at the concentration tested, HBsAg was not involved in the HBV-mediated suppression of IFN-α in pDCs and PBMC.

Since we previously demonstrated that circovirus DNA can suppress TLR9-mediated activation of pDCs [19], we next investigated whether the impairment of TLR9-induced IFN-α may directly involve neutralising CpG sequences within the HBV DNA itself. For this purpose, 400 complete HBV genome sequences [20] were analysed for their CpG content and in particular for CpG-containing hexamers, known to suppress “classical” CpG-mediated immune activation [21]. Our results suggest that a majority of CpG motifs within HBV genome display a neutralising structure pattern (CCG, CGG, CGCG) [21], ranging from 59 to 68% between different HBV genotypes (Table 1). More importantly, we identified the presence of three well known neutralising hexamers (GCGGCG, CGCGGG and GCCGTT [21]) in nearly all HBV genotypes (Table 1). We then synthetized oligonucleotides derived from HBV DNA containing these neutralising motifs (A*T*CCTG CGC GGG ACGTCC*T*T as ODN HBV; * standing for phosphorothioate link). ODNHBV1 was found to inhibit CpG 2216-induced IFN-α secretion in a dose-dependent manner, with 31%, 35% and 41% of inhibition respectively at 1:1, 2:1 and 3:1 ratio (Figure 2D). In conclusion, our data suggest that i) HBV virions suppress the TLR9-mediated secretion of IFN-α and IL-6 in pDCs and B cells respectively, without any effect on TLR7 signaling and that ii) HBV DNA sequences negatively affects TLR9-induced IFN-α.

**Table 1:** Percentages of neutralising CpG dinucleotides and CpG-containing hexamer frequencies within HBV genotypes and subgenotypes.

Genotype^a^	subgenotype	Total CG^b^	CCG^c^	CGCG^d^	CGG^e^	% CpG-N^f^	GCCGTT	GCGGCG	CGCGGG
A	A1	100	28	4	27	59		1	
	A2	103	29	6	28	61	1	1	
B	B1	101	32	4	28	63		2	1
	B2	105	35	5	30	67		2	1
	B3	108	37	4	31	67		2	1
	B4	109	36	5	30	66		2	1
C	C1	106	30	5	33	64	2	2	1
	C2	103	33	4	31	66	2	2	1
	C3	100	27	4	33	64	1	1	
	C4	103	31	5	30	64	1	1	
D	D1	101	31	4	27	61	1	2	1
	D2	102	31	4	27	61	1	2	1
	D3	101	30	4	27	60	1	2	1
	D4	101	33	4	26	62	1	2	1
E		99	33	3	30	63	1	1	
F	F1	103	29	4	30	61	1	1	
	F2	101	31	4	32	66	1	2	1
G		97	33	3	26	64	1	1	
H		101	32	4	32	67	1	1	

a analysis of 400 complete HBV genome sequences was carried out using previously reported HBV sequences [20] and additional data base (H. Norder).

b frequency of total CpG within HBV genome.

c,d,e frequency of CpG with a neutralising structure pattern [21] within HBV genome.

f pourcentage of neutralising CpG within HBV genome calculated as follow f = (c+d+e)*100/b.

### HBV virions down regulate TLR9 mRNA and protein expression in PBMC

**Figure 3:**
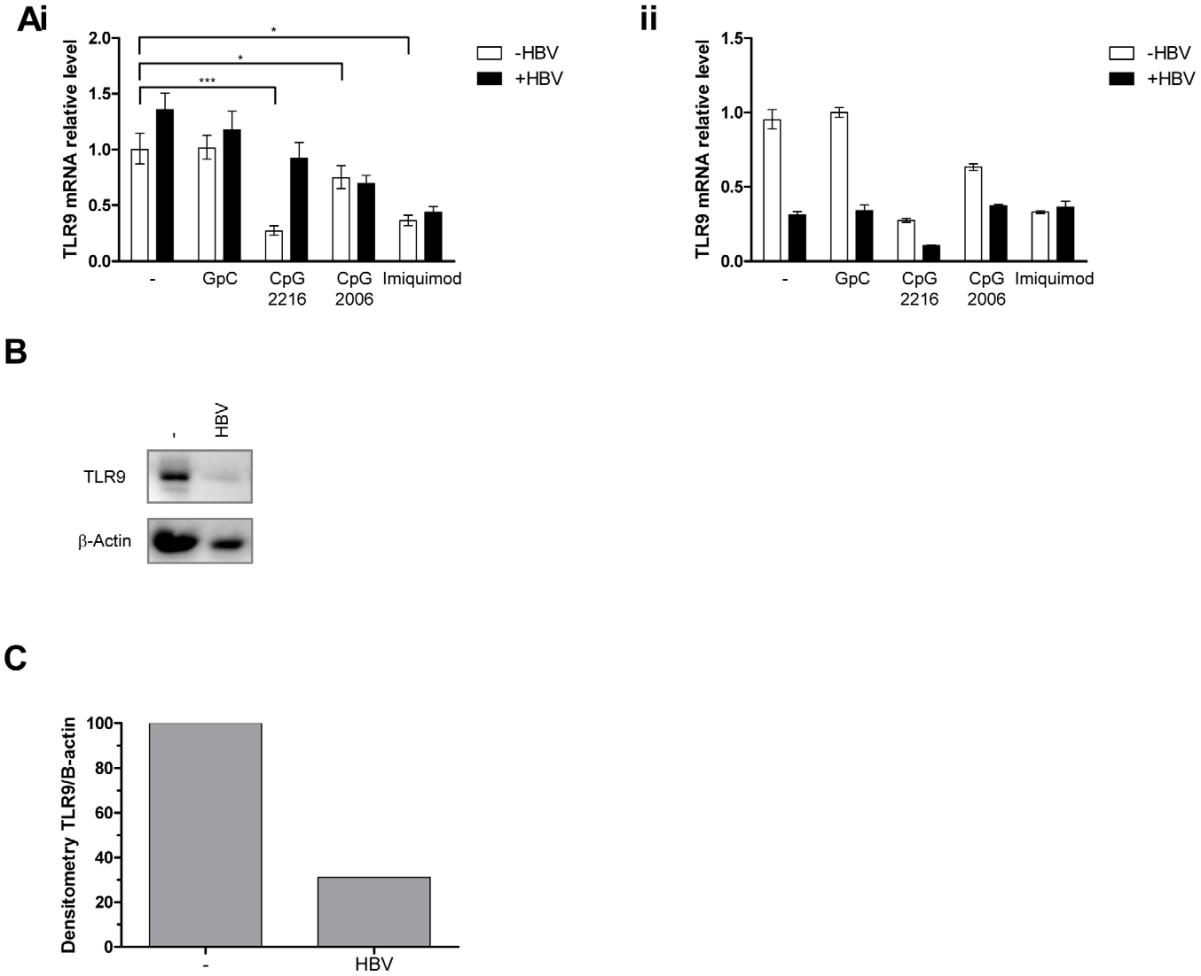
TLR9 mRNA and protein levels are down regulated in the presence of HBV virions in PBMC. (**A**) HBV down regulates TLR9 mRNA. PBMC were stimulated with the indicated TLR ligands ± HBV (MOI 100) for 7 hours (Ai) or 24h (Aii). Cells were harvested for RNA extraction and RT-PCR reaction. The expression level of TLR9 was assessed by qPCR, normalized to the β2-microglobulin expression and expressed relative to untreated cells. Results are representative of 1 of 5 experiments that gave similar results using >3 different blood donors and 2 independent HBV stocks. (B) TLR9 protein levels are reduced by HBV virions. PBMC were incubated with HBV or left untreated for 24h. TLR9 expression level was assessed by immunoblotting and β-actin immunodetection was performed as a loading control. Protein levels were assessed through densitometry with Fuji Multigauge software. (C) Results are representative of 1 of 2 experiments that gave similar results using two different blood donors and 2 independent HBV stocks.

We previously demonstrated that dsDNA oncoviruses such as HPV16 and EBV down regulate TLR9 transcription [22], [23]. According to *ex vivo* analysis of down regulation of TLR9 transcripts in PBMC from HBV-infected individuals [24], we analyzed *in vitro* the kinetic of down regulation of TLR9 transcripts in PBMC stimulated with HBV virions. For this purpose, TLR9 mRNA levels were determined by qPCR in cells stimulated with CpG or the TLR7 ligand imiquimod ± HBV virions for 7h and 24h. As shown in Figure 3Ai, a 7h treatment with CpG 2216, CpG2006 or imiquimod down regulated TLR9 mRNA levels (unpaired Student *t* test compared with NT; p < 0.001, p < 0.05, and p < 0.05 respectively). Interestingly, HBV increased TLR9 mRNA levels upon CpG 2216 stimulation. By contrast, at 24h, HBV strongly down-regulated TLR9 mRNA expression in untreated PBMC and upon both CpG stimulation of pDCs (CpG 2216) and B cells (CpG 2006) (Figure 3Aii). As shown in Figure 3 (B, C), the inhibition of TLR9 mRNA levels correlated with a reduced TLR9 protein expression in PBMC treated with HBV for 24h. In accordance with the observation that TLR7 signaling was not affected by HBV (supplementary figure S2A and B), TLR7 mRNA levels were similar to untreated and CpG2216 treated cells (supplementary Figure S2C), demonstrating that TLR7 expression is not impaired by HBV virions. Thus HBV specifically down regulated TLR9 transcription and protein levels in PBMC.

### The TLR9 promoter is down regulated by HBV virions

**Figure 4:**
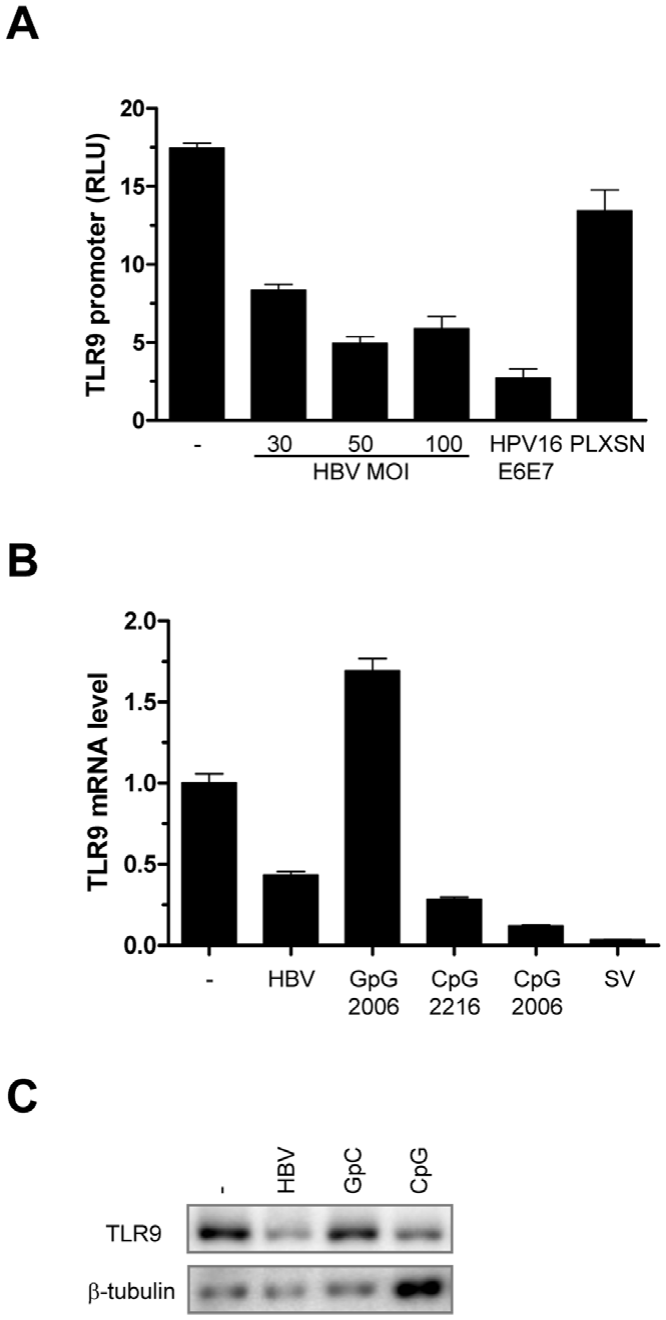
TLR9 is down regulated at the promoter, mRNA and protein level by HBV virions. (A)*TLR9* promoter activity is reduced in RPMI 8226 B cells in the presence of HBV. RPMI 8226 cells were transfected with the *TLR9* promoter, incubated with HBV virions at the indicated MOI or left untreated for 24h and harvested in total 48h post transfection. Co-transfection of RPMI 8226 cells with the *TLR9* promoter and HPV16E6E7 or pLXSN constructs was used as positive and negative controls respectively. Cells were then processed as previously described for luciferase activity [22]. (B) *TLR9* mRNA levels are reduced in RBMI 8226 cells in the presence of HBV virions. Cells were harvested 24h after treatment for RNA extraction and RT-PCR reaction. The expression level of *TLR9* was assessed by qPCR, normalized to the β2-microglobulin levels and expressed relative to untreated cells. (C) HBV alters the TLR9 protein levels in RPMI 8226 cells. Cells were treated as indicated and protein extracts were prepared 24h later. TLR9 protein level was assessed by immunoblotting. β-tubulin served as a loading control. Graphs shown are representative of 1 of 5 experiments that gave similar results using 3 batches of HBV virus.

HBV virions may decrease TLR9 levels by two distinct mechanisms: HBV can affect the stability of TLR9 transcript or can directly inhibit the TLR9 transcription as we observed for HPV16 and EBV [22], [23]. To discriminate between these two possibilities, we have determined whether HBV virions can influence the activity of the TLR9 promoter. The isolated TLR9 promoter cloned in front of luciferase reporter gene was introduced by transient transfection in the B cell line RPMI8226, which expresses high levels of endogenous TLR9, as previously described [22]. We observed that 24h post-treatment with HBV (MOI ranging from 30-100), the TLR9 promoter was suppressed (Figure 4A), as previously shown with HPV E6/E7 protein expression [22]. Furthermore both mRNA and protein expression of TLR9 in RPMI8226 B cells were down regulated when treated for 24h hours with HBV virions (Figure 4B and C), confirming our findings regarding B cells from PBMC . Thus another strategy used by HBV to block TLR9 regulated pathways is to down regulate TLR9 transcription.

### HBV virions block IRF7-mediated induction of IFNα4

**Figure 5:**
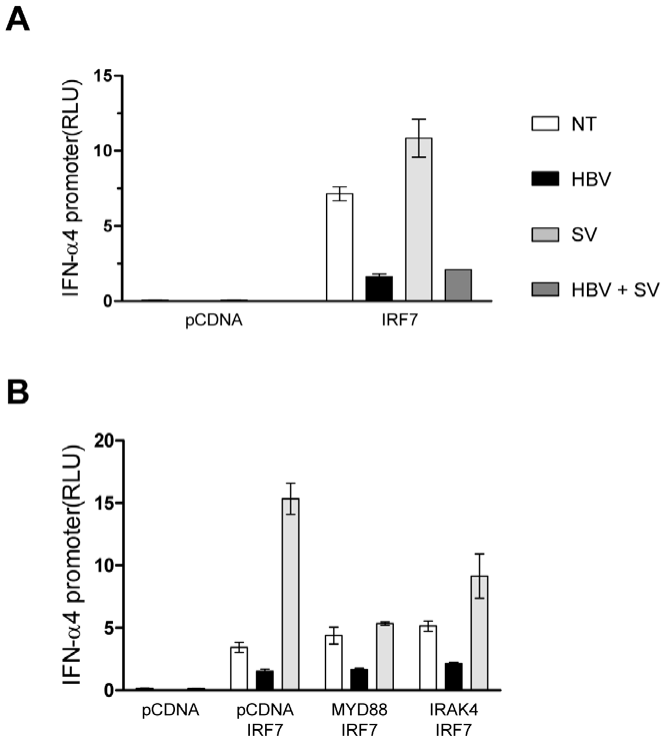
The IRF7-mediated induction of the IFNα4 promoter is suppressed by HBV. (A) Co-transfection of the *IFNα4* promoter (500 ng) with the IRF7 expression plasmid or the control vector (750 ng) was performed in HEK293. After 24h cells were incubated with HBV (MOI 100) and/or SV and luciferase activity was measured post total 48h. (B) HEK293 cells were co-transfected with the expression vectors indicated (750 ng) with the *IFNα4* promoter (500 ng). After 24h cells were treated as described above and luciferase activity was measured post 48h. Values represent the mean of three independent experiments performed in triplicate.

It has been recently reported that HBsAg inhibited TLR9-mediated IRF7 expression and nuclear translocation, which are important for induction of IFN*-α* gene [16]. In our model, TLR9 induction of IFN-α in pDCs and PBMC was blocked using HBV virions (Figure 2). TLR9 is known to activate two signaling pathways dominated by the transcription factors IRF7 and NF-κB. The IRF7-mediated pathway of signal transduction is responsible for the transcription of IFN-α and IFN-β. As shown in Figure 5A, expression of IRF7 in HEK293 cells led to the induction of the IFNα4 promoter linked to a luciferase reporter gene, which was further increased by infection with Sendai virus (SV), a ssRNA virus known to induce the RIG-I-IRF7 signaling pathway [25], [26]. Addition of HBV virions to the cells blocked luciferase activity, arguing for an impairment of the IRF7 pathway (Figure 5A). Furthermore we observed that SV-enhanced induction of the IFNα4 promoter by IRF7 was also blocked by HBV virions (Figure 5B). In order to determine where in the TLR9 pathway HBV exerts its inhibitory effect, we co-transfected IRF7 with the IFNα4 promoter ± MyD88 or IRAK4 constructs in HEK293 cells. We observed that in all cases, HBV down regulated IRF7-mediated induction of the IFNα4 promoter ± MyD88 or IRAK4 (Figure 5C). MyD88 or IRAK4 alone did not induce the IFNα4 promoter (Supplementary figure S3). These data indicate that HBV acts downstream of MyD88 and IRAK4 and upstream of IRF7 to block IFN-α production.

### Chronic HBV carriers (CHB) and hepatocellular carcinoma patients (HCC) display a reduced TLR9 expression

**Figure 6:**
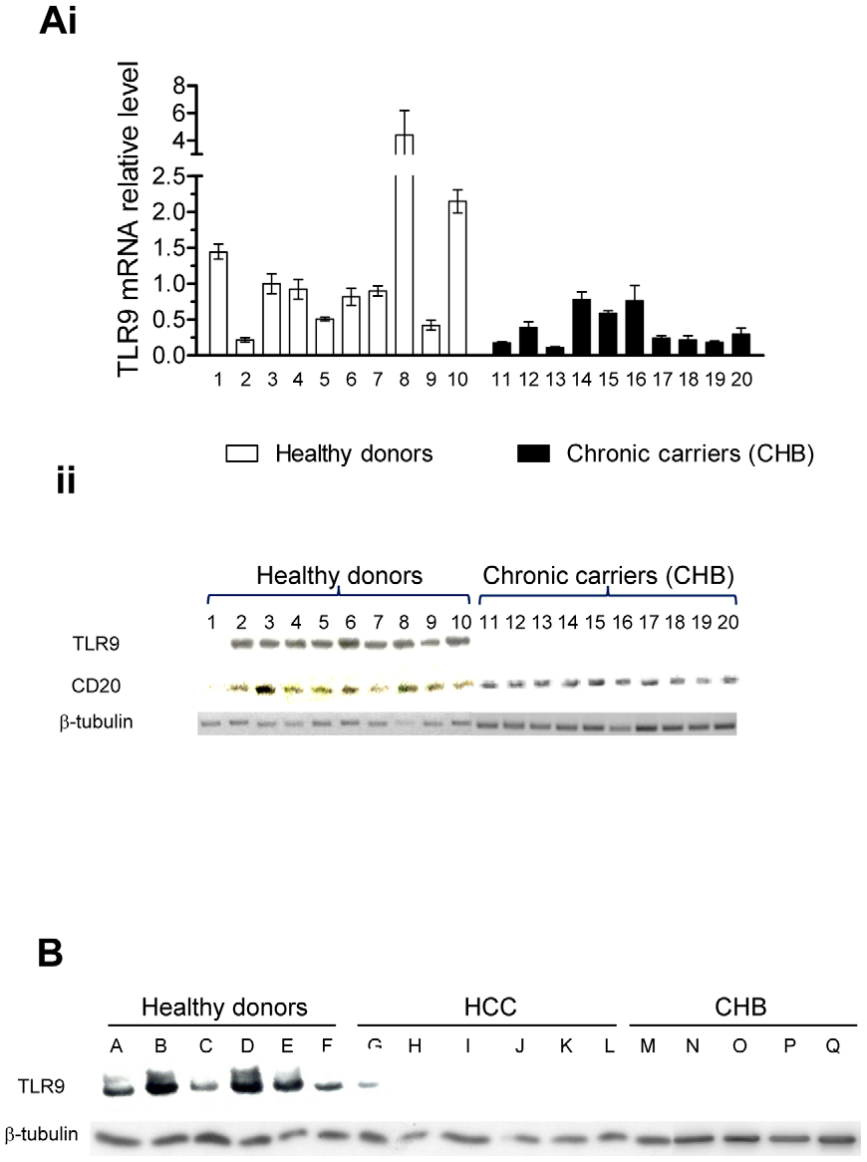
TLR9 mRNA and protein expression is impaired in CHB and HCC patients. (A) CHB patients present a reduced TLR9 mRNA and protein level. PBMC were processed for RNA extraction (Ai) and protein extraction (Aii). TLR9 mRNA expression level was assessed in qPCR, normalized to β2-microglobulin and expressed relative to an healthy donor (sample number 3). TLR9 and CD20 protein levels were determined by immunoblotting and β-tubulin served as a loading control. (B) TLR9 protein expression is inhibited in CHB and HCC patients. Buffy coats were processed for protein extraction. TLR9 and β-tubulin (the loading control) were detected by immunoblotting.

To corroborate our *in vitro* data we isolated PBMC from ten European chronic hepatitis B patients and ten healthy blood donors (Table 2) and analyzed TLR9 mRNA and protein levels. CHB patients displayed an overall reduced TLR9 mRNA expression in comparison to controls (Figure 6i). Interestingly, TLR9 protein was not detectable in CHB patients, reflecting a drastic shut down of TLR9 in both pDCs and B lymphocytes, the latter being identified by CD20 staining (Figure 6ii). In addition, TLR9 protein levels were also analysed in buffy coats from Asian individuals, that consisted in 5 CHB, 6 HBV-associated HCC patients and 5 hospital-based controls (table 2). TLR9 protein was also drastically reduced in CHB and HCC patients (Figure 6B). These results show that TLR9 protein levels are drastically decreased in peripheral pDCs and B cells from patients with HBV-related liver disease.

**Table 2:** Characteristics of patients with chronic hepatitis B (CHB), HBV-related hepatocellular carcinoma (HCC) and healthy controls from European and Asian origins.

European patients (n)	Age (year) M/SD	gender	HBeAg	HBsAg (IU/ml)	Viremia (IU/ml)
Controls (10 ; 1 to 10)	32,3 /12,5	5M - 5F	NEG	NEG	NEG
CHB patients (10)					
Patient 11	36	M	NEG	861,2	undetectable
Patient 12	60	F	NEG	96,8	undetectable
Patient 13	59	F	NEG	38,6	undetectable
Patient 14	43	M	NEG	430,5	511
Patient 15	28	F	NEG	5817,5	2,4.10^6^
Patient 16	32	F	POS	>25000	48.10^6^
Patient 17	51	F	NEG	5328,8	undetectable
Patient 18	50	M	NEG	104,9	undetectable
Patient 19	57	F	NEG	1416	2550
Patient 20	66	M	NT	NT	undetectable
**Asian patients (n)**	**Age (year)**	**gender**	**HBeAg**	**HBsAg**	**Viremia (DNA copie/ml)**
Controls (6 ; A to F)	54,8 /8,4	6M	NEG	NEG	NEG
HCC patients (6)					
Patient G	44	M	NT	POS	5,6.10^6^
Patient H	47	M	NT	POS	25.10^6^
Patient I	53	M	NT	POS	3,7.10^6^
Patient J	42	M	NT	POS	2,7.10^6^
Patient K	61	F	NT	POS	79.10^6^
Patient L	59	M	NT	POS	22.10^6^
CHB patients (5)					
Patient M	58	F	NT	POS	0,76.10^6^
Patient N	56	F	NT	POS	1,8.10^6^
Patient O	50	M	NT	POS	4,8.10^6^
Patient P	57	F	NT	POS	5,5.10^6^
Patient Q	58	F	NT	POS	23.10^6^

CHB, chronic hepatitis B ; HCC, hepatocellular carcinoma; NT not tested; Age M, median; SD standard deviation; All patients are negative for hepatitis C infection.

## Discussion

Increasing evidences are highlighting the role of innate immunity, and TLR in particular, following HBV infection and during the course of chronic hepatitis [27], [28], [29]. This study provides new insights into the ability of HBV to modulate TLR9 signaling in pDCs and B cells and to inhibit TLR9-mediated IFN-α and IL-6 secretion. Unlike many viruses which trigger type I IFNs and have evolved to counteract this activation [30], our data indicate that HBV does not trigger IFN-α secretion in pDCs although it can impair its function by a selective TLR9 signaling blockade. The poor levels of HBsAg glycosylation [31] may account for the absence of pDC sensing since glycosylation appears to be a key element for glycoprotein-mediated IFN induction in pDCs [32]. Our findings show that, *in vitro*, HBV virions can inhibit CpG-induced IFN-α in PBMC and purified pDCs from naïve individuals, arguing for a direct role of virus-mediated IFN-α suppression, in correlation with the reduced IFN-α response following *ex vivo* stimulation of pDCs from chronic HBV carriers [10], [12], [13], [15]. We report that HBV can block TLR9-regulated pathways by down regulating TLR9 transcription, reducing TLR9 mRNA and protein levels in total PBMC. Such inhibition was also observed in RPMI8226, a B cell line expressing TLR9 [22], and since TLR9 levels in PBMC reflect both pDC and B cell expression, our data suggest for the first time that HBV can also inhibit TLR9 signaling and transcription in B cells.

Since circulating DCs do not support HBV replication [18], and given the ability of pDCs to sense viruses in the absence of replication, we hypothesised that not only HBV virions, but also HBV secreted antigens, such as HBsAg, may interfere with CpG-induced IFN-α secretion. Under our experimental conditions, up to 1µg of recombinant HBsAg did not affect IFN-α secretion. However, since HBV replication is associated with the secretion of tremendous amounts of subviral particles in the blood of HBV-infected individuals [5], we cannot exclude that higher amounts of HBsAg or preS1/preS2 antigens may impair TLR9-induced IFN-α, as recently shown by Xu *et al* using 25 µg of serum-derived HBsAg [16]. HBsAg was shown to inhibit TLR9-mediated IRF7 expression and nuclear translocation, which are important for induction of IFN-α gene expression [16]. Here we showed that HBV virions block transcription of the IFNα4 promoter induced either by triggering the MyD88- IRAK4-IRF7 axis or by Sendai virus infection. We believe that HBV exerts its inhibitory effect on IFNα4 promoter by directly targeting IRF7. Of note, Epstein-Barr and Kaposi’s sarcoma viruses have also been shown to down-regulate IFN-α response in pDCs by blocking IRF7 [30], [33].

In addition, we have identified previously characterized neutralising CpG motifs [21], [34] in nearly all HBV genotypes. Krieg *et al* [21] first reported the existence of neutralising CpG motifs in adenovirus DNA that can block CpG-mediated immune stimulation. Further studies have characterized the suppressive effects of specific CpG motifs or G and GC-rich ODN on TLR9 activation [35], [36]. The identification of neutralising motifs within HBV DNA suggests that the HBV genome may be involved in TLR9 silencing in a competitive manner. However, an immunoregulatory role for HBV DNA on the TLR9-IFN axis during chronic HBV infection is presently unknown.

Moreover, in a previous study, we demonstrated that E6 and E7 from human papillomavirus 16 and LMP-1 from EBV have the ability to downregulate the TLR9 promoter and halter its functionality [22], [23]. Here, we also observed that HBV can reduce the levels of TLR9 promoter and consequently mRNA transcripts. We have shown that HPV16 and EBV activate the NF-κB pathway which in turns acts as a negative regulator of TLR9 transcription. Many studies have shown that HBx can play opposite roles regarding the activation of NF-κB [29]. However a role for HBx in down regulating TLR9 in pDCs is unlikely since they do not support HBV gene expression. One may argue that a putative candidate could be the HBV polymerase since it has recently been shown to inhibit the transcription of the IFN-β gene in hepatocytes by inhibiting the interaction between IKKε and DDX3 DEAD box [28]. Overall, distinct mechanisms may be involved in hepatocytes and pDCs, which, respectively, do not express TLR9 and are not permissive to HBV infection [18].

By contrast to the inhibition of TLR9-mediated IFN-α secretion, HBV had no effect on TLR7-induced IFN-α in pDCs. Similarly, HIV gp120, HCV virions and HPV E6/E7 proteins have also been shown to suppress TLR9-mediated IFN-α induction without any effect on TLR7 signaling [22], [37], [38]. Thus both TLR pathways might differ at yet unidentified levels and our results support the hypothesis of a more stringent control of TLR9 expression in comparison to that of TLR7 [17], which may protect the host from an overproduction of IFN-α but may also facilitate virus persistence.

Analysis of PBMC from chronic hepatitis B and HBV-associated hepatocellular carcinoma patients suggest that TLR9 mRNA levels are downregulated in CHB and that TLR9 protein is drastically reduced in both CHB and HCC patients. These findings corroborate our *in vitro* data in naïve PBMCs stimulated with HBV. Such a substantial reduction in TLR9 expression in chronic HBV carriers has also been observed by others [39]. A role for HBsAg in the inhibition of TLR9-mediated IRF7 expression and nuclear translocation has been reported [16]. However we have not been able to confirm this hypothesis neither *in vitro*, nor with the *ex vivo* analysis since the number of CHB patients (10) studied was not sufficient to address potential correlations between HBsAg quantification and TLR9 mRNA levels.

Overall, this study demonstrates that HBV virions can selectively inhibit TLR9 expression in pDCs and B lymphocytes from PBMC isolated from naïve and CHB patients. Such inhibition is associated with a functional loss of TLR9-regulated pathways. Although the biological significance of HBV-induced TLR9 impairment remains unclear, recent data suggest that TLR9 stimulation may induce liver damage (inflammation, fibrosis, steatosis), involving non-parenchymal liver components (stellate, Kupffer and sinusoidal endothelial cells) [40]. In this context, HBV-mediated TLR9 inhibition may reduce liver inflammation thereby facilitating its persistence within the liver. Therefore, understanding TLR-HBV interactions holds future promise not only for the fundamental aspects of HBV persistence but also for the development of novel immunotherapeutic strategies for chronic hepatitis B.

## Materials and Methods

### PBMC and pDCs isolation

Human blood from healthy subjects was obtained from the French blood agency (Lyon, France). PBMC were separated by centrifugation on lymphoprep (Abcys). pDCs were either positively selected with anti-BDCA-4-conjuguated magnetic microbeads or negatively selected using Plasmacytoid Dendritic Cell Isolation Kit (Miltenyi Biotec). Purity of sorted pDCs (85 to 95%) was analysed by staining with anti-BDCA-2 and anti-CD123 monoclonal antibodies (Miltenyi Biotec) by flow cytometry (FACSCAN). Viability of stimulated pDCs was assessed using the Annexin-V-FLUOS Staining Kit (Roche Applied Science). PBMC from chronic HBV carriers (European samples) and from Asian HBV-positive patients (CHB and HCC) were obtained with informed consent from each patient with the procedure approved by the local Ethics Committee.

### *In vitro* stimulation of PBMC and pDCs

Cells were cultured in RPMI 1640 (Life Technologies) supplemented with 10% fetal calf serum (Hyclone), 2 mM L-glutamine (Invitrogen) and antibiotics (Penicillin-streptomycin, Life Technologies). Freshly isolated PBMC and purified pDCs were cultured at 5x10^6^ cells/ml and 10^5^ cells/ml respectively. Cells were stimulated with the TLR9 ligands CpG2006, CpG2216, the control GpC2006 or GpC2116 at 2µM (Invivogen,). TLR7 stimulation was performed using 3 distinct ligands, loxorubine at 1mM, resiquimod at 1µM (TLR7/8) and imiquimod at 4µM (Invivogen). Supernatants were collected after stimulation at indicated time points and cytokines (IFN-α; IL-6 and IL-10) were determined using specific ELISAs with respective sensitivity of 8; 1,5; 1,6 and 3,1pg/ml (Abcys).

### Hepatitis B virus, recombinant antigens and HBV-derived ODN

HBV inoculum was a 100-fold concentrated culture supernatant from HepG2.2.15 cells or HBV infected-HepaRG cells [41], [42]. Briefly, supernatants were clarified, layered onto a sucrose cushion (10 and 20% in 20 mM Tris, pH 8) and centrifuged at 4°C for 16h at 25,000 rpm using a SW41 rotor (Beckman). HBV inoculum was also obtained following concentration using Centricon Plus-70 (Biomax 100, Millipore Corp). Similar procedure was applied to HepG2 or HepaRG cells supernatants to generate the mock lysate. HBV stock titre (genome equivalent/ml, GEq/ml) was assessed using real time PCR [43]. Recombinant HBsAg, adr (0,69mg/ml) and ayw3 (0,88mg/ml) subtypes, were provided by BioMérieux [44]. Endotoxin levels in recombinant HBsAg were less than 0,01 EU/ml within HBV stocks (Limulus amoebocyte lysate assay, BioWhittacker). HBV-derived ODN (table 2) were synthetised by Eurogentec, resuspended in endotoxin-free water and used at 2µM. Sendai virus (SV, Cantell strain; *Charles River*, SPAFAS) was added directly to the cells to give a final concentration of 80 hemagglutinin (HA) units/ml.

### Electron and confocal microscopy

pDCs were prepared for electron microscopy as previously described [45] and immunogold labelling was performed using a rabbit anti-HBsAg antibody (MA Petit, INSERM U871). Observations were performed on a JEOL JEM 1400 transmission electron microscope (Jeol Ltd) equipped with Gatan Orius camera and Analysis Soft Imaging System (Eloïse SARL). For confocal microscopy, purified pDCs were fixed with 4% paraformaldehyde in PBS for 20 min at room temperature. Cells were permeabilized in 0,5% saponin with 5% BSA for 30 min at room temperature. Cells were washed in 0,1% saponin/PBS and incubated with primary rabbit anti-HBsAg antibody for 2h at room temperature followed by incubation with secondary goat anti-rabbit Alexa fluor 488 (Invitrogen Life Technologies) performed for 1h at room temperature. After mounting with Cytomation medium (DAKO), cells were examined using a Pascal 510 ZEISS LSM microscope and imaged using Zeiss LSM Image Browser.

### RT-qPCR and semi-qPCR

Total cellular RNA was extracted from PBMC using the RNA extraction kit (Qiagen) or the protein/RNA extraction kit (Macherey-Nagel). cDNA synthesis was performed with 1 µg of total RNA with the First Strand cDNA Synthesis kit (MBI Fermentas). qPCR was performed using the Mx3000P real-time PCR system (Strategene) with Mesa green qPCR MasterMix plus (Eurogentec) and TLR9 and β2µglobulin specific primers for relative quantification [22], [23].

The expression level of human TLR7 was determined by semi-qPCR using TLR7 and GAPDH primers. Each reaction was run in duplicate.

### Constructs

The Expression vector for human IRF7 plasmid construct and the human IFNα4 promoter cloned in front of *firefly* luciferase (IFNα4-PGL3) were kindly provided by Kate Fitzgerald (UMass Medical School, MA, USA). The -3227TLR9-pGL3 reporter plasmid, the retroviral vector HPV16E6E7 and the control pLXSN have been previously described [22]. The expression vector for MyD88 and for IRAK4 have been previously described.

### Transient transfection and luciferase assay

RPMI 8226 and HEK293 cells have been transiently transfected in triplicate using Fugene 6 (Roche Applied Science) and Gene Juice (Novagen) respectively and luciferase assay was performed as previously described [22].

### Immuno-blot analysis

Total protein extracts were obtained with the protein/RNA kit (Macherey-Nagel) following manufacturer’s instructions. Twenty µg of protein extracts were quantified with the protein quantification assay (Macherey-Nagel). Antibodies used for immunoblotting were TLR9 (Cell Signaling), β-tubulin (Sigma), β-Actin (MP-biomedicals) and CD20 (BD Pharmingen). Western blots were developed using the intelligent dark box (Fuji film).

### Statistics

GraphPad (version 5) was used to calculate unpaired and paired *p* values: ***, extremely significant, *p* < 0.001; **, very significant, *p*  =  0.001-0.01; *, significant, *p*  =  0.01-0.05; and *ns*, *p* > 0.05.

## Supporting Information

Figure S1 **Suppressed** **IFN-α production is not caused by increased pDC apoptosis.** pDCs were treated with CpG 2216 ± HBV at MOI 100 or 50 for 12h. Cells were stained with Annexin V^FITC^ by flow cytometry and percentages in gate R1 represent early apoptotic pDCs.
(TIF)

Figure S2 **HBV does not impair TLR7-induced IFN-α and does not modulate TLR7 transcripts.** pDCs were stimulated with TLR7 ligand loxoribine (Lox) at 1mM (A) or TLR7/8 ligand resiquimod (Res) at 1µM (B) ±HBV at MOI 50 or 100. Supernatants were collected after 24h and tested for IFN-α (pg/ml) by ELISA. Experiments were performed on cells isolated from 3 different blood donors. (C) HBV does not modulate TLR7 mRNA in pDCs. PBMC were stimulated with CpG 2216 or GpC2216 in presence of mock lysate or HBV at MOI 50. After 20h, cells were harvested for RNA extraction, and RT-PCR was performed for TLR7 and GAPDH expression. Densitometry levels were determined using the Bio-Rad phosphoimaging software. Results are representative of 3 different blood donors and 2 independent HBV stocks.
(TIF)

Figure S3 **Over-expression of IRF7 in HEK293 cells leads to IFNα4 promoter activation.** Cells were co-transfected with IFNα4-pGL3 reporter plasmid together with the indicated plasmids. After 48h luciferase assay was assessed as described previously [20] .
(TIF)
