# Supplementary figures and images for: Hepatitis B Virus Impairs TLR9 Expression and Function in Plasmacytoid Dendritic Cells
Source: PLoS One. 2011 Oct 25;6(10):e26315. doi: 10.1371/journal.pone.0026315 (PMC3201951; doi:10.1371/journal.pone.0026315)

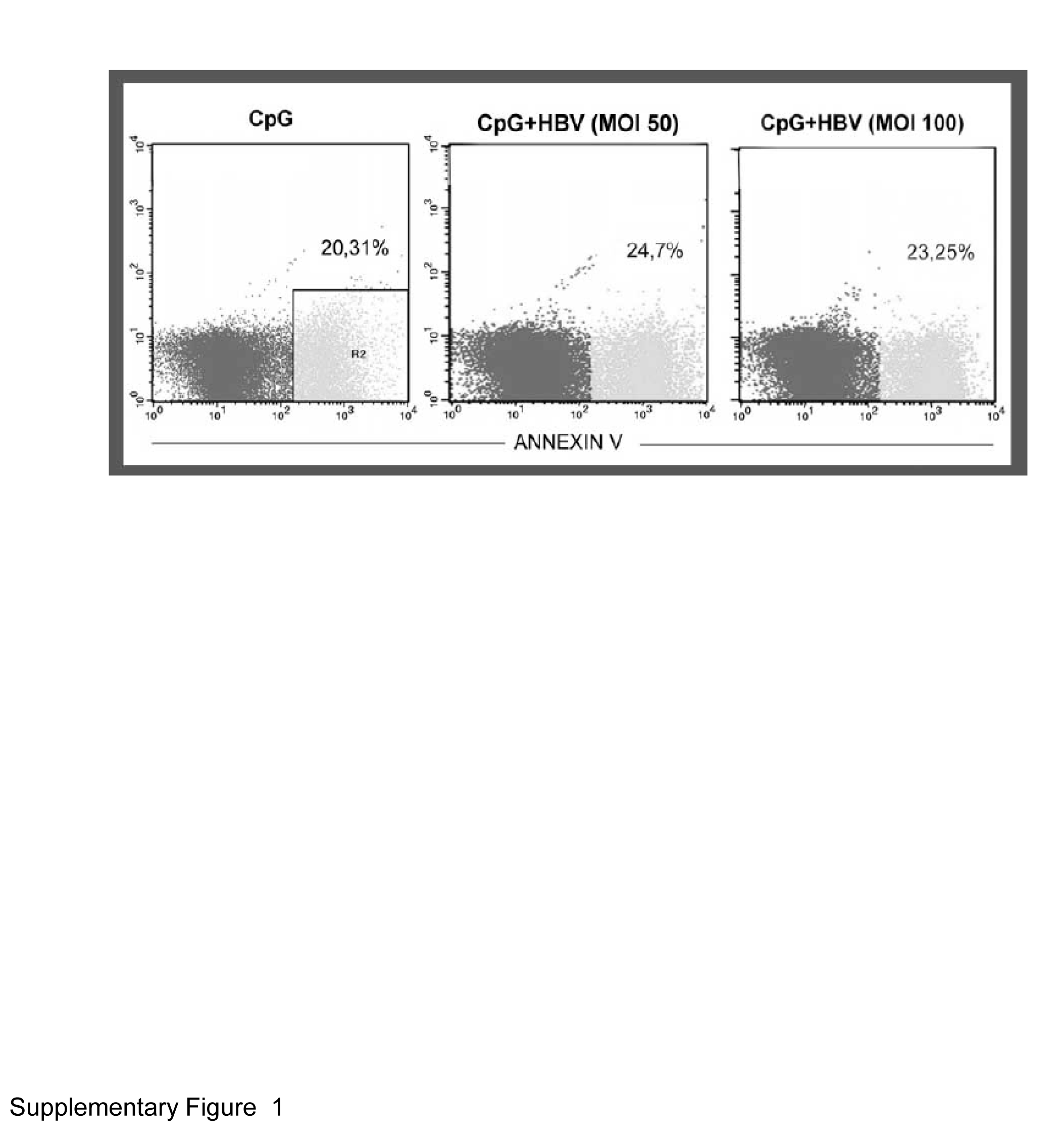

Supplement: Figure S1 — Suppressed IFN-α production is not caused by increased pDC apoptosis. pDCs were treated with CpG 2216 ± HBV at MOI 100 or 50 for 12h. Cells were stained with Annexin VFITC by flow cytometry and percentages in gate R1 represent early apoptotic pDCs. (TIF) [file pone.0026315.s001.tif]

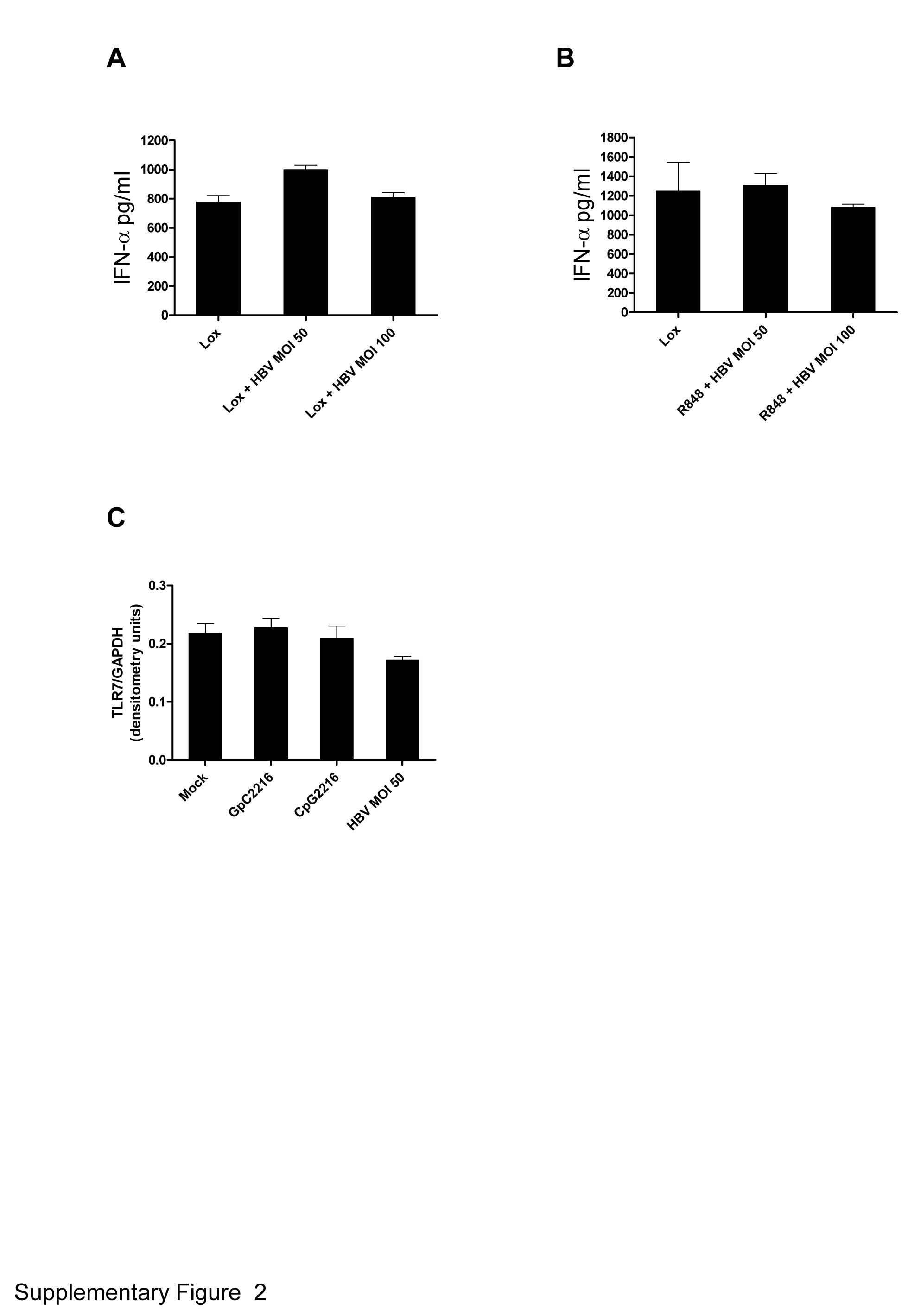

Supplement: Figure S2 — HBV does not impair TLR7-induced IFN-α and does not modulate TLR7 transcripts. pDCs were stimulated with TLR7 ligand loxoribine (Lox) at 1mM (A) or TLR7/8 ligand resiquimod (Res) at 1µM (B) ±HBV at MOI 50 or 100. Supernatants were collected after 24h and tested for IFN-α (pg/ml) by ELISA. Experiments were performed on cells isolated from 3 different blood donors. (C) HBV does not modulate TLR7 mRNA in pDCs. PBMC were stimulated with CpG 2216 or GpC2216 in presence of mock lysate or HBV at MOI 50. After 20h, cells were harvested for RNA extraction, and RT-PCR was performed for TLR7 and GAPDH expression. Densitometry levels were determined using the Bio-Rad phosphoimaging software. Results are representative of 3 different blood donors and 2 independent HBV stocks. (TIF) [file pone.0026315.s002.tif]

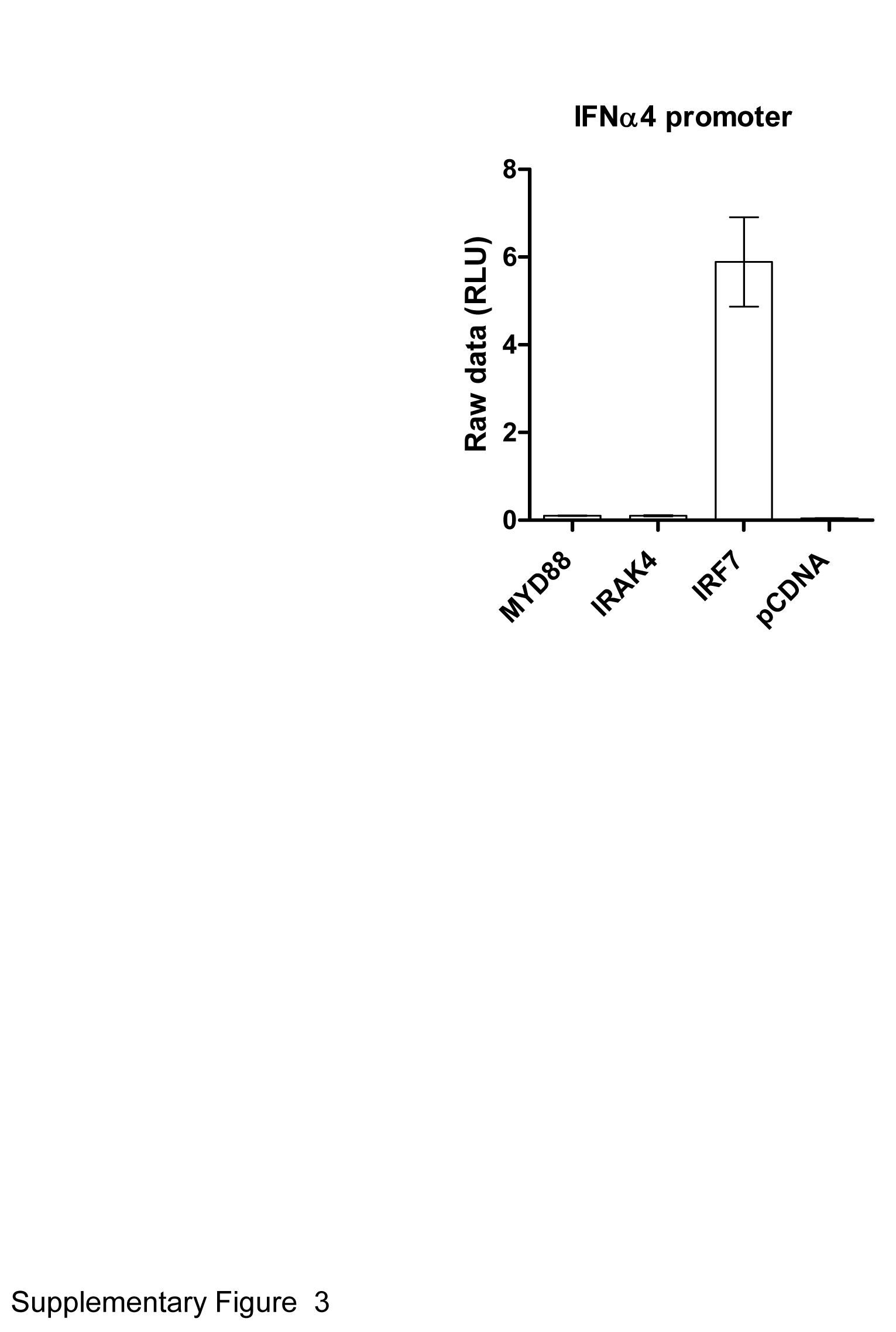

Supplement: Figure S3 — Over-expression of IRF7 in HEK293 cells leads to IFNα4 promoter activation. Cells were co-transfected with IFNα4-pGL3 reporter plasmid together with the indicated plasmids. After 48h luciferase assay was assessed as described previously [20] . (TIF) [file pone.0026315.s003.tif]
